# ECLS in Pediatric Cardiac Patients

**DOI:** 10.3389/fped.2016.00109

**Published:** 2016-10-07

**Authors:** Matteo Di Nardo, Graeme MacLaren, Marco Marano, Corrado Cecchetti, Paola Bernaschi, Antonio Amodeo

**Affiliations:** ^1^Pediatric Intensive Care Unit, Children’s Hospital Bambino Gesù, Rome, Italy; ^2^Cardiothoracic Intensive Care Unit, National University Health System, Singapore; ^3^Paediatric Intensive Care Unit, Department of Paediatrics, The Royal Children’s Hospital, University of Melbourne, Parkville, VIC, Australia; ^4^Microbiology Unit, Children’s Hospital Bambino Gesù, Rome, Italy; ^5^ECMO and VAD Unit, Children’s Hospital Bambino Gesù, Rome, Italy

**Keywords:** ECMO, neonates, children, VAD, heart failure

## Abstract

Extracorporeal life support (ECLS) is an important device in the management of children with severe refractory cardiac and or pulmonary failure. Actually, two forms of ECLS are available for neonates and children: extracorporeal membrane oxygenation (ECMO) and use of a ventricular assist device (VAD). Both these techniques have their own advantages and disadvantages. The intra-aortic balloon pump is another ECLS device that has been successfully used in larger children, adolescents, and adults, but has found limited applicability in smaller children. In this review, we will present the “state of art” of ECMO in neonate and children with heart failure. ECMO is commonly used in a variety of settings to provide support to critically ill patients with cardiac disease. However, a strict selection of patients and timing of intervention should be performed to avoid the increase in mortality and morbidity of these patients. Therefore, every attempt should be done to start ECLS “urgently” rather than “emergently,” before the presence of dysfunction of end organs or circulatory collapse. Even though exciting progress is being made in the development of VADs for long-term mechanical support in children, ECMO remains the mainstay of mechanical circulatory support in children with complex anatomy, particularly those needing rapid resuscitation and those with a functionally univentricular circulation. With the increase in familiarity with ECMO, new indications have been added, such as extracorporeal cardiopulmonary resuscitation (ECPR). The literature supporting ECPR is increasing in children. Reasonable survival rates have been achieved after initiation of support during active compressions of the chest following in-hospital cardiac arrest. Contraindications to ECLS have reduced in the last 5 years and many centers support patients with functionally univentricular circulations. Improved results have been recently achieved in this complex subset of patients.

## Introduction

Mechanical circulatory support is an important tool in the management of children with cardiac failure. Two major forms of mechanical circulatory support are currently available in neonates and children: extracorporeal life support (ECLS, also known as extracorporeal membrane oxygenation or ECMO) and ventricular assist device (VAD). Each of these devices has advantages and disadvantages. Another device that has been used in older children is the intra-aortic balloon pump. As mechanical circulatory support in children has evolved, the indications have expanded and outcomes have improved.

**Figure 1:**
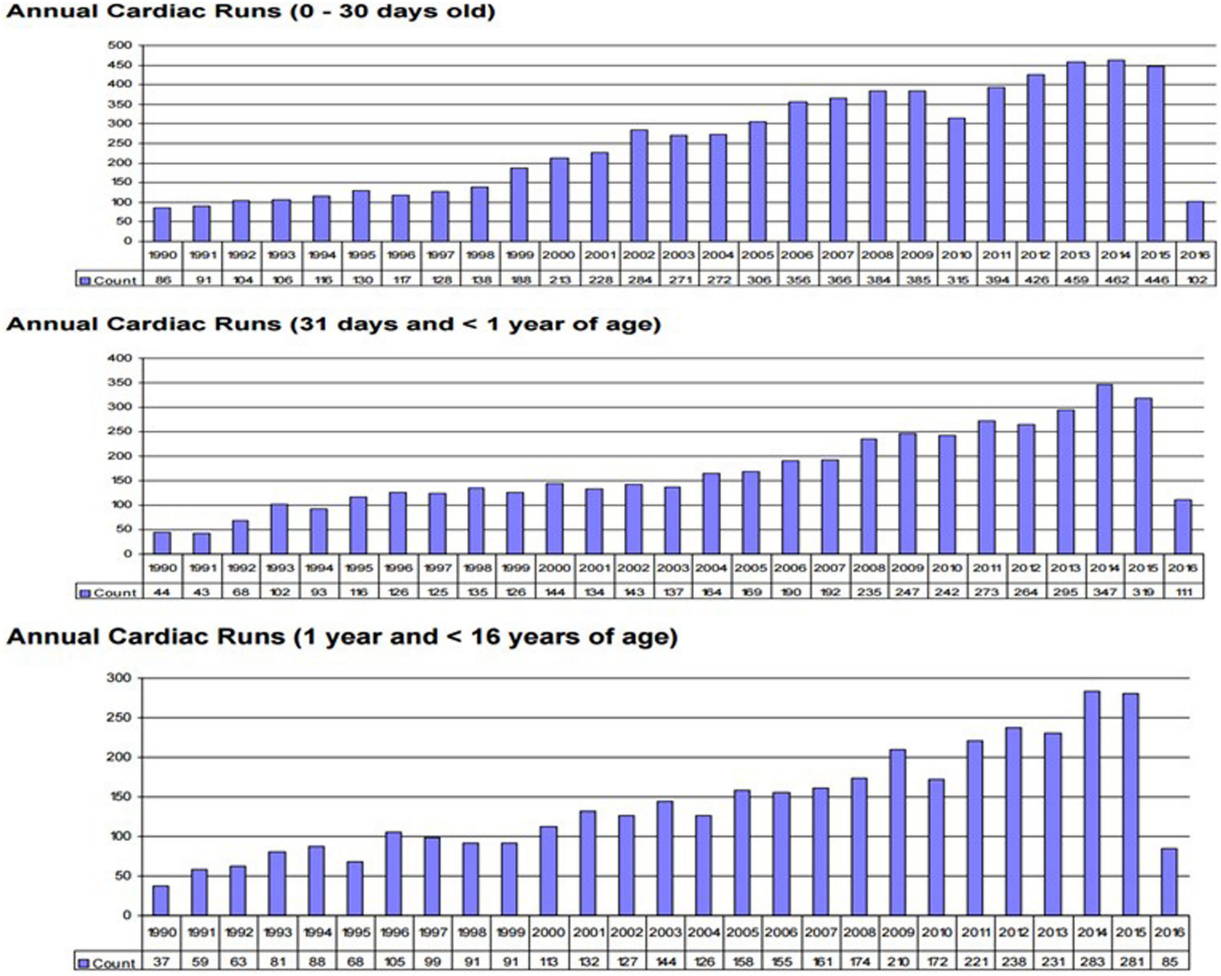
**Annual neo/ped ECMO runs**. (International Summary 2016. Courtesy of P. Rycus from the ELSO Registry).

**Figure 2:**
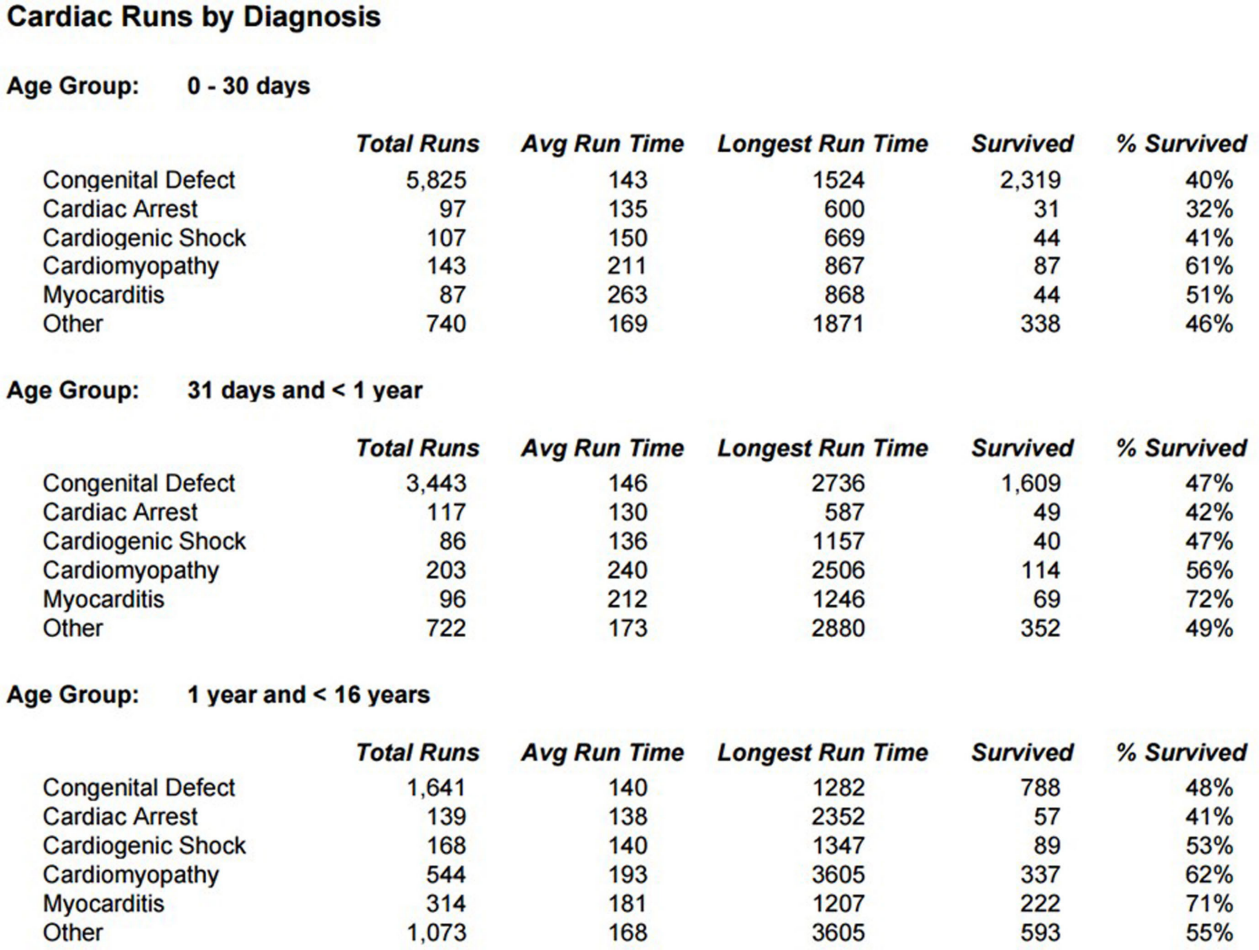
**Cardiac Runs by diagnosis: % of survival on ECMO**. Avg, average.

Survival for children with heart failure on ECLS has progressively improved over the past two decades, and this is occurring despite placing more complex patients on support, including a significant proportion with single-ventricle physiology, end-stage heart failure or in cardiac arrest (1). Survival to hospital discharge after cardiac ECLS is generally between 40 and 50% (2, 3). However, it is important to consider not only crude, short-term survival but also the quality of survival in terms of functional outcome. Unfortunately, data regarding quality of life after cardiac ECLS are generally limited to single center experiences and insufficient research has taken place to study this in greater detail. Data from the Extracorporeal Life Support Organization (ELSO) Registry indicate that ECLS was used to provide cardiac support for over 1,200 pediatric patients in 2016 (Figure 1) (1). Important potential determinants of outcome after ECLS for cardiac disease can be broadly categorized according to indication (the underlying disease); timing of ECLS initiation; center-specific strategies (cannulation), as well as comorbidities in individual patients (Figure 2).

The purpose of this review article is to outline current management strategies in the application of ECLS to children with heart disease, review recent evidence about the effect of these strategies, and provide an overview of the topic for clinicians.

## Indications for Cardiac ECLS

Indications and contraindications for ECLS in neonates and children with cardiovascular disease have evolved during the past 45 years. Advances in ECLS circuit design and greater recognition that ECLS is beneficial for indications beyond early postoperative support have resulted in steady growth in the number of pediatric patients who receive cardiac ECLS. The indications for ECLS can be divided into two groups: those involving cardiac surgery and not. The indications related to cardiac surgery include preoperative stabilization, failure to wean postcardiotomy, low cardiac output syndrome in the postoperative period, and cardiac arrest. The indications in the absence of cardiac surgery are cardiac arrest, myocarditis and cardiomyopathy, pulmonary hypertension, intractable arrhythmias, and other forms of shock, such as sepsis or Kawasaki’s Disease (4).

### Preoperative Stabilization

Extracorporeal life support is required in a small subset of neonates prior to initial surgical repair or palliation. The primary goals of ECLS support are to optimize the hemodynamic state, maximize oxygen delivery, and prevent or mitigate any multiorgan failure prior to surgical repair. The use of ECLS in this setting may improve the candidacy of some patients with complex surgical repair. Hypoxemia and cardiogenic shock in patients with arterial transposition or hypoplastic left heart syndrome with inadequate atrial shunting have been the most common indications for preoperative ECLS support (4). Neonates with late presentation arterial transposition and pulmonary hypertension represent a high-risk population. ECLS may be necessary to provide cardiopulmonary stabilization until pulmonary vascular resistance declines and the arterial switch operation can be safely performed (5–7). Neonates with absent pulmonary valve syndrome who present with refractory respiratory failure despite aggressive mechanical ventilation may also be candidates for preoperative stabilization, although the associated respiratory complications are often fatal in the long-term despite this (8). Similarly, patients with severe Ebstein’s anomaly and functional pulmonary atresia who present with low cardiac output secondary to a circular shunt, intractable dysrhythmias, or cyanosis secondary to inadequate pulmonary blood flow may benefit from VA ECLS stabilization during the period of transitional circulation until pulmonary vascular resistance declines and a stable source of pulmonary blood flow is established (9, 10).

### Perioperative Support

The role of ECMO to provide postcardiotomy support for children with severe cardiac dysfunction after surgery for congenital heart disease (CHD) is well established. Mechanical circulatory support may be required in the postoperative period, either due to inability to wean from bypass or because of a progressive low cardiac output syndrome. Patients who are subjected to prolonged periods of cardiopulmonary bypass can experience severe postoperative myocardial dysfunction that may prevent successful separation from cardiopulmonary bypass. Children who have preexisting ventricular dysfunction or certain structural cardiac abnormalities [such as hypoplastic left heart syndrome (HLHS)] may be at greatest risk. Once correctable residual anatomic lesions have been excluded, ECLS may be used to provide short-term cardiopulmonary support. In most cases, the cannulation strategy utilized for cardiopulmonary bypass is adequate during and after the transition to ECMO. Veno-arterial ECMO is the most commonly used modality in neonatal and pediatric cardiac patients during the perioperative period although veno-venous ECMO may be used in select circumstances, such as intraoperative pulmonary hemorrhage, which can occasionally occur in lesions, such as Tetralogy of Fallot and its related variants.

There is no consensus regarding the optimal timing for ECMO initiation in the perioperative period. Some reports suggest the absence of a clear relationship between timing of ECLS initiation and clinical outcome, whereas others describe improved survival in patients who receive early ECLS support, including those who transition to ECLS in the operating room, compared to those who receive support later in the postoperative period (11–16). ECMO is used to provide perioperative support in 10% patients with HLHS who undergo the Norwood operation (17). Irrespective of the patient population, it is important to recognize that earlier initiation of ECLS facilitates unloading of vulnerable myocardium, prevention or reduction of acidosis, and reduced risk of cardiovascular collapse.

### ECLS Beyond the Perioperative Period

Although the vast majority of neonatal and pediatric cardiac ECLS occurs in patients with structural CHD who are undergoing surgical repair or palliation, patients without structural heart disease may require ECLS to manage severe heart failure.

Children with acute fulminant myocarditis can benefit from mechanical circulatory support as a bridge to recovery. Cardiomyopathy (8.3%) and myocarditis (4.5%) are the most common non-structural etiologies of heart failure in neonates and children reported to the ELSO registry (1). Overall survival in this patient population is ~67% and appears to be greatest in older pediatric patients and in those without signs of end-organ injury, such as renal failure (18, 19). Data suggest that ECMO may be necessary in ~50% of patients who present with myocarditis (20). Low cardiac output state and arrhythmias are the primary indications for ECLS in the majority of these patients. Rhythm-related low cardiac output states may result from tachycardia-induced cardiomyopathy or the myocardial depressant effects of anti-arrhythmic medications (21). ECLS is also equal occasionally used to provide support for patients who experience cardiovascular collapse due to accidental or intentional poisoning (22) and for patients who experience life-threatening pulmonary hypertensive crises (23). The use of mechanical cardiac support in the management of end-stage heart failure has dramatically evolved during the past two decades. Short-term extracorporeal devices and durable implantable VADs are now standard heart failure therapies in adults and children. Technologic advances in highly efficient implantable centrifugal and axial flow pumps have proven difficult to miniaturize for use in infants and neonates. Consequentially, ECLS remains an important component of mechanical heart failure therapy in some patient populations.

Extracorporeal life support can be used as a bridge to transplantation in children with irreversible myocardial dysfunction (24–26). This includes patients with dilated cardiomyopathy, restrictive cardiomyopathy, and end-stage congenital cardiac disease. However, data from a large, propensity score-matched study showed that overall survival rates are significantly higher in pediatric patients who are bridged to transplantation with a VAD than ECLS (27). Even if VAD appears to be a superior form of bridge to transplantation in older children and in children with non-structural heart disease, VAD technology in small children has higher complication rates than in older children and adults, and outcomes can be disappointing (28). In one study, 29% of children experienced a stroke and 97% experienced some form of serious adverse event during support (28). There is general consensus that ECLS is useful as a *bridge-to-decision* and as a *bridge-to-bridge* (bridge-to-VAD) (29). When used in the setting of acute hemodynamic decompensation, including extracorporeal cardiopulmonary resuscitation (ECPR), ECLS is readily available and considerably less expensive compared to VAD. In most cases, the degree of myocardial recovery, if any, is unknown at time of initiation therefore ECLS is preferred. During this period of clinical uncertainty, ECLS is useful as a bridge to decision about transplant candidacy because neurologic tests and organs (liver and kidney) recovery can be evaluated.

Extracorporeal life support can also be used after heart transplantation. Indications for support in this setting include failure to separate from cardiopulmonary bypass and progressive low cardiac output syndrome in the immediate postoperative period, along with circulatory collapse due to rejection or graft vasculopathy later in the course (30, 31). Overall survival is approximately 50% in this population (1) and neonates and infant seem to be at greater risk for death than older children. The use of ECLS as a bridge to recovery for patients who experience severe acute rejection remote from transplantation is less successful and many patients will ultimately require re-transplantation (32). Right heart failure and/or pulmonary hypertension are often responsible for the inability to separate from cardiopulmonary bypass and ECLS can be very useful in managing this postoperatively.

## Contraindications for Cardiac ECLS

The list of contraindications to ECLS in children with heart disease is shrinking over time. Absolute contraindications include lethal chromosomal abnormalities, severe irreversible brain injury, and extremely low gestational age and weight (<32 weeks gestation or <1.5 kg). Relative contraindications include moderate intra-ventricular hemorrhage, gestational age <34 weeks, weight <2.0 kg, and certain high-risk congenital heart lesions, such as those that co-exist with congenital diaphragmatic hernia. Data from the ELSO registry indicate that the overall survival rate for premature infants (<37 weeks gestation) who receive cardiac ECLS is 31%, compared to 41% survival in term infants (1). Similarly, the reported survival rate following ECPR approaches 30% in preterm babies and is as low as 21% in neonates <34 weeks gestational age (33). Weight <2 kg has historically also been a relative contraindication to ECLS but survival a rate of 10% has been reported in neonates with hypoplastic left heart syndrome weighing <2.5 kg when placed on ECLS (34) and a survival rate of 33% has been reported for infants weighing <3 kg at time of cardiac ECLS support (35).

## Neonatal and Pediatric Cannulation

The approach to cannulation should be flexible and based on the underlying need for ECMO. Transthoracic cannulation of the right atrial appendage and the ascending aorta can be used in cases of failure to wean from cardiopulmonary bypass. In the immediate postoperative period, reopening the sternal wound and direct cardiac cannulation provides the most expeditious route to institute support, especially in patients who suffer cardiac arrest. Adequate venous drainage and excellent arterial perfusion should be assured by chest cannulation, providing the cannulas are placed properly; however, severe hemorrhage and mediastinitis remain disadvantages of chest cannulation, making peripheral cannulation preferable in most other settings.

Cannulation of the right internal jugular vein and the common carotid artery provides excellent venous drainage and perfusion and is the preferred cannulation site in neonates and children below 15 kg. Cannulation of the femoral vessels provides adequate venous drainage and perfusion for larger children. A second venous drainage cannula placed in the right internal jugular vein may be added if venous drainage is inadequate through the femoral route. To avoid ischemia of the lower extremities, a perfusion cannula in the distal femoral artery is placed by many groups. Venous congestion of the lower extremities is less frequent and usually does not require treatment.

In cases of severe heart dysfunction, inadequate decompression of the left-sided cardiac chambers during ECLS may be seen. A number of strategies can be implemented to address this. Left-sided distension may be managed by increasing ECLS flow to empty the right heart, minimizing pulmonary blood flow, and decreasing pulmonary venous return to the left heart. However, this increases the afterload to the systemic ventricle, which may cause the aortic valve to close, in turn risking thrombus formation in the ventricle as well as hemorrhagic pulmonary edema if the end-diastolic pressure rises too high. In cases of persistent distension of the systemic ventricle, a balloon atrial septostomy can be performed in the cardiac catheterization laboratory. If this is not technically possible, additional surgical options include changing to central cannulation, with or without biatrial cannulation, inserting an atrial vent via a pulmonary vein, or inserting an apical ventricular vent (36). These vents are incorporated into the ECLS circuit by means of a Y-connector.

## Special ECLS Indications

### Intractable Arrhythmias with Hemodynamic Compromise and Procedural Support

In selected patients with malignant tachyarrhythmias or bradyarrhythmias, ECLS may be necessary to mitigate cardiogenic shock while medical therapy is being optimized or in order to safely facilitate catheter ablation or pacemaker insertion. Generally, patients with severe myocarditis or dilated cardiomyopathy are supported with ECLS to manage severe arrhythmias. Severe arrhythmias can also present after cardiac surgery or in lesions, such as Ebstein’s malformation (37–39). Catheter-based interventional and diagnostic procedures can be safely performed on patients receiving ECLS support (40). The most common indications for cardiac catheterization on ECLS are assessment of operative results and percutaneous left heart decompression. Early detection and correction of residual cardiac lesions is associated with improved survival so the use of catheter-based diagnostic procedures should be considered when non-invasive diagnostic studies fail to identify a reason for inability to separate from ECLS (41, 42). ECLS has also been considered as a safe alternative to cardiopulmonary bypass during complex airway surgery and repair of pulmonary artery sling (43).

### Extracorporeal Cardiopulmonary Resuscitation

As the experience with ECLS has grown, new indications have evolved, including emergent resuscitation. del Nido and colleagues (44) initially described the use of rapid resuscitation with ECLS after cardiopulmonary arrest. In a retrospective study, all patients with CPR duration of less than 15 min survived, while only just over half survived when those times were greater than 42 min (45). Clinical outcomes tend to be better in children who require ECPR for underlying cardiac disease than those who have cardiac arrest without structural heart disease (46). This may be because children with structural heart disease who suffer cardiac arrest are often monitored in an intensive care setting and have adequate vascular access. These patients tend to be younger, less likely to have preexisting organ dysfunction, and more likely to experience ventricular dysrhythmias than asystole prior to cardiac arrest (47, 48). Potential benefits of ECPR in these patients include improved myocardial oxygen delivery, reduced myocardial workload, reduced vasopressor and inotropic therapy, reduced pulmonary barotrauma and intrathoracic pressure, improved end-organ perfusion and oxygen delivery, reversal of acidosis, and targeted temperature control. Current resuscitation guidelines from the International Liaison Committee on Resuscitation support the use of ECPR in pediatric patients with a cardiac diagnosis who experience in-hospital cardiac arrest in a center with ECLS expertise (49).

### ECLS in Patients with Functionally Univentricular Circulation

Many centers previously considered a functionally univentricular circulation a contraindication to ECLS. However, this view is anachronistic and outcomes can be satisfactory in many instances. In addition to the same indications for ECLS as in children with biventricular hearts, shunt thrombosis may also require ECLS and is associated with excellent outcomes when the thrombosis is swiftly identified and corrected. However, while the indications are similar, the cannulation strategies require careful planning.

**Figure 3:**
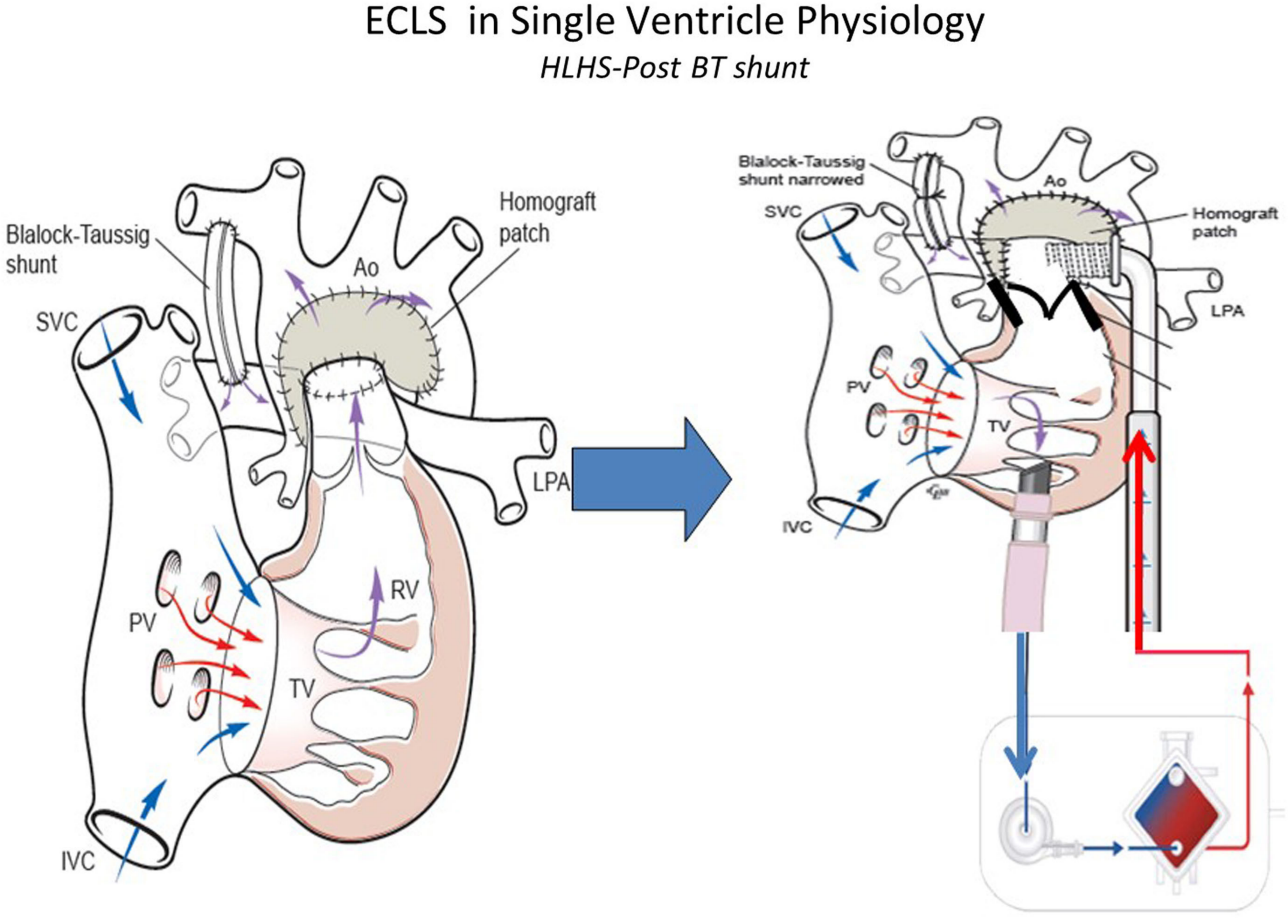
**ECLS in single-ventricle physiology: first stage support with Blalock–Taussig (BT) shunt using ECMO or VAD according to patient’s gas exchange**. Technical aspects: to better support first stage hypoplastic left heart syndrome the BT shunt should be narrowed to avoid pulmonary overflow. SVC, superior vena cava; IVC, inferior vena cava; PV, pulomanry veins; TV, tricuspidal valve; RV, right ventricle; LPA, left pulmonary artery; RPA, right pulmonary artery; Ao, aorta (courtesy of Dr. Massimo Griselli).

Management of the infant with single ventricular physiology and a systemic-pulmonary shunt can be safely achieved by leaving the shunt patent and increasing the net ECLS circuit flow to compensate for the pulmonary run-off (Figure 3). Generally a 150–200 ml/kg/min of blood flow may be required. This management associated with better outcomes than occluding the shunt, which was often done historically an attempt to reduce pulmonary edema from excessive pulmonary blood flow (50, 51). In situations where the lungs are normal, it is possible also to remove the oxygenator and continue ECLS as a centrifugal VAD. Sherwin and colleagues (14) reported the outcomes of a large multicenter cohort of neonates requiring ECMO after stage 1 palliation for HLHS. These authors used the data of the ELSO Registry from 2000 to 2009 to evaluate the survival to hospital discharge in this subset of patients. Among 738 neonates, the survival was 31%. Thus, the mortality for neonates with HLHS supported with ECMO was high compared with those with other defects (14). This study showed how longer ventilation time before ECMO, longer support duration and ECMO complications, such as renal failure, myocardial stun, inotrope requirement, metabolic acidosis, and neurologic injury, increase the mortality of these patients.

There is limited experience with mechanical support in patients with cavo-pulmonary connections and outcomes are not so good as for other indications, most likely due to a combination of previous surgery, complex physiology, difficult cannulation approach, the need to address venous drainage from both superior and inferior vena cava and the inadequacy of conventional cardiopulmonary resuscitation (CPR) to provide sufficient blood flow if these children suffer of cardiac arrest (52–54).

**Figure 4:**
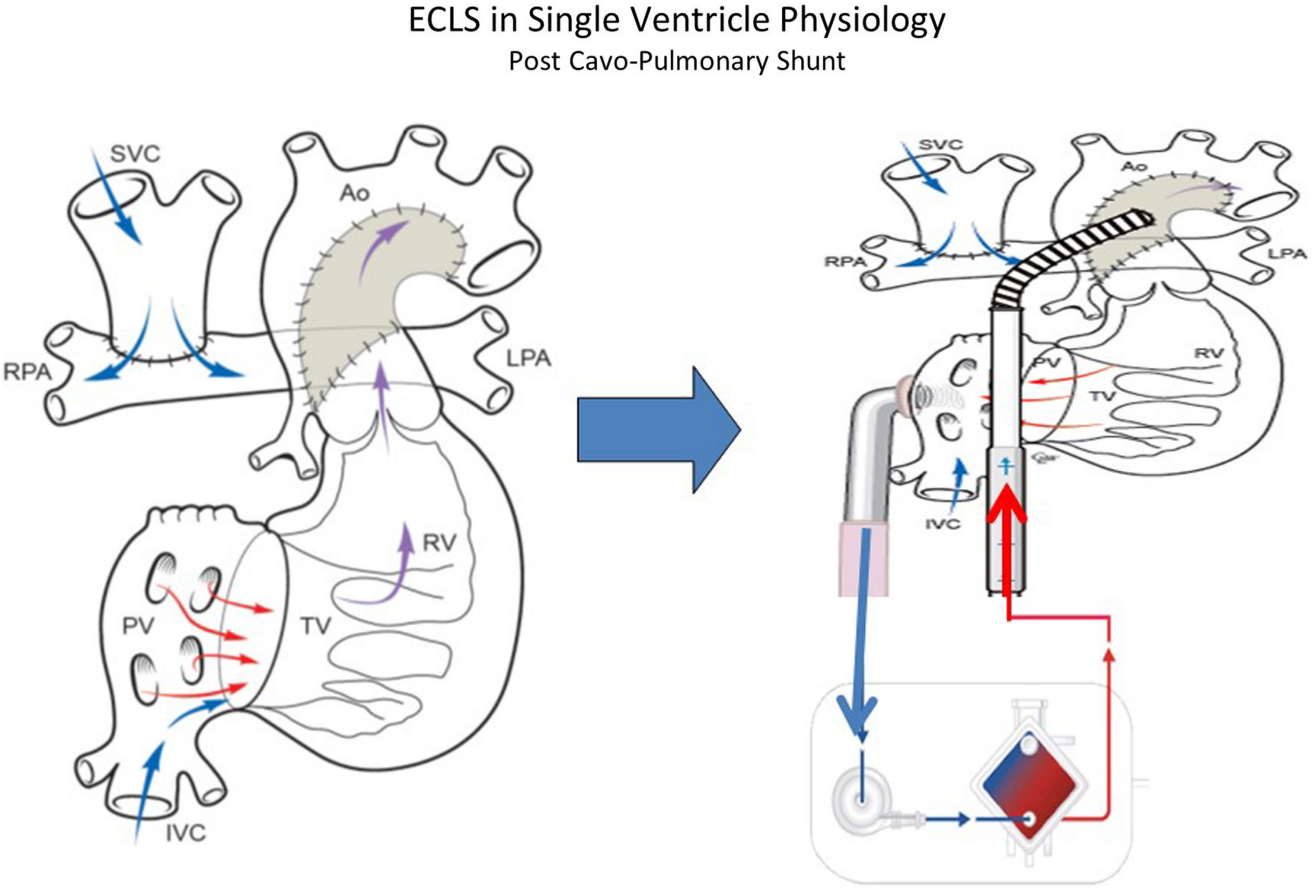
**ECLS in single-ventricle physiology: second stage support (Glenn Physiology) using ECMO or VAD according to patient’s gas exchange**. Technical consideration: the inflow cannula can be atrial or ventricular (courtesy of Dr. Massimo Griselli).

Patients who have undergone Glenn procedure (Figure 4) have a unique cardiopulmonary-cerebral physiology that limit the success of CPR and ECMO. Jolley and colleagues (52) analyzed the data of the ELSO Registry from 1999 to 2012 to evaluate the survival to hospital discharge in children with Glenn physiology. A total of 103 infants were identified and survival to hospital discharge was 41%. Survival is high compared with the outcome data from similar ELSO Registry analysis following stage I palliation for HLHS (31%) and Fontan (35%). The unique surgical anatomy and physiology of Glenn procedure may explain the limited efficacy of standard medical management and resuscitation. The approach to ECLS in patients who have the Glenn procedure is particular. In these patients, there are two separated source of venous return to the single ventricle: the IVC carrying desaturated blood directly to the heart and the superior vena cava (SVC), which indirectly returns oxygenated blood, via the lungs, to the common atrium. An inability to maintain adequate venous drainage and systemic perfusion may contribute to worse outcomes. When dual venous cannulae are placed (femoral/atrial and SCV), Booth and colleagues (55) recommend placing the SVC cannula first to address cerebral venous congestion. In multivariate analysis (52), the need for inotropic support before ECMO, longer duration of ECMO and the development of renal failure remained statistically significant in increasing mortality. Combined cardiopulmonary indication for ECMO was also statistically significant when analyzing for mortality, suggesting that the combination of both respiratory and cardiac disease in this subset of patients is difficult to manage with ECMO. These indicators of poor prognosis could aid in clinical management decisions regarding the utility and/or timing of initiation of ECMO.

**Figure 5:**
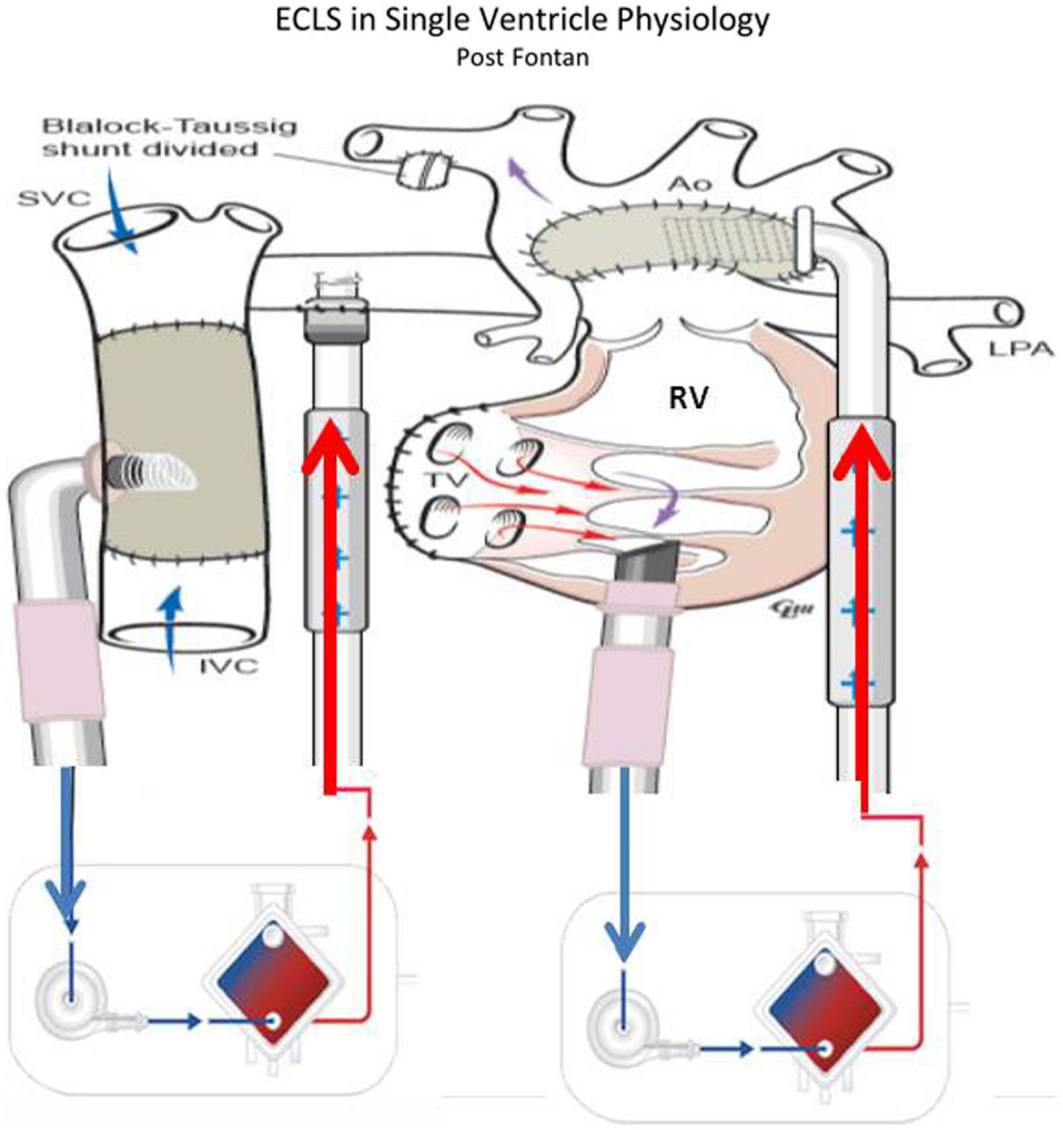
**ECLS in single-ventricle physiology: third stage support (Fontan Physiology) using ECMO or VAD according to patient’s gas exchange (courtesy of Dr. Massimo Griselli)**.

Adequate flow drainage is complicated also in Fontan patients (53, 54). Due to the difficulty of transthoracic cannulation in this population, peripheral cannulation may be used, resulting in inadequate decompression of either the superior or inferior vena cava unless cannulation of both the femoral and internal jugular veins is performed. The trade-off is that with adequate cavo-pulmonary decompression, preload to the single ventricle is decreased due to reduced blood flow through the lungs. The single ventricle is often failing, and imposing high afterload from external arterial flow can inhibit ventricular ejection of any blood traversing the pulmonary bed (Figure 5). Together these factors can create a state where most of the cardiac output and oxygen delivery must be provided by the ECMO flow, with little or no contribution from ventricular ejection; however, fully supportive flows often cannot be achieved with peripheral cannulation alone. In these patients with Fontan physiology, CPR is generally ineffective and despite the challenges associated with ECMO, elective cannulation and initiation of ECMO before cardiac arrest in patients with potentially reversible causes of a failing Fontan has been used (53, 55). In the largest series of 230 patients, 35% of all patients with Fontan procedure on ECMO survived to hospital discharge. In this subset, CPR prior to ECMO initiation was associated with non-survival. Mortality also was associated with the duration of ECMO support and the development of renal failure or neurological complications (54, 55).

## Conclusion

Many forms of MCS are available for children with heart failure refractory to conventional management. Actually, ECMO can be used in many settings to support pediatric patients with cardiac disease. However, the use of ECMO in this field requires careful selection of patients and timing of interventions remains challenging. The principles used presently in the application of ECMO to support the failing circulation in children will be used as the foundation for developing innovative circulatory techniques for the future.

## Author Contributions

Dr. MN and Dr. MM wrote 80% of the manuscript. Dr. CC and Dr. PB wrote the remaining 20% of the manuscript. Dr. GM and Dr. AA revised the manuscript.

## Conflict of Interest Statement

The authors declare that the research was conducted in the absence of any commercial or financial relationships that could be construed as a potential conflict of interest.
